# Perioperative platelet reactivity over time in patients undergoing vascular surgery: An observational pilot study

**DOI:** 10.1371/journal.pone.0304800

**Published:** 2024-06-26

**Authors:** A. R. T. Brand Kanters, N. C. Roozendaal, N. M. J. Parr, G. Pasterkamp, R. T. Urbanus, S. J. A. Korporaal, Gert J. de Borst

**Affiliations:** 1 Department of Vascular Surgery, University Medical Center Utrecht, Utrecht University, Utrecht, The Netherlands; 2 Clinical Diagnostics Laboratory, University Medical Center Utrecht, Utrecht University, Utrecht, The Netherlands; 3 Van Creveldkliniek, University Medical Center Utrecht, Utrecht University, Utrecht, The Netherlands; BSMMU: Bangabandhu Sheikh Mujib Medical University, BANGLADESH

## Abstract

**Background:**

Despite Antiplatelet therapy (APT), cardiovascular patients undergoing revascularisation remain at high risk for thrombotic events. Individual response to APT varies substantially, resulting in insufficient protection from thrombotic events due to high on-treatment platelet reactivity (HTPR) in ≤40% of patients. Individual variation in platelet response impairs APT guidance on a single patient level. Unfortunately, little is known about individual platelet response to APT over time, timing for accurate residual platelet reactivity measurement, or the optimal test to monitor residual platelet reactivity.

**Aims:**

To investigate residual platelet reactivity variability over time in individual patients undergoing carotid endarterectomy (CEA) treated with clopidogrel.

**Methods:**

Platelet reactivity was determined in patients undergoing CEA in a prospective, single-centre, observational study using the VerifyNow (change in turbidity from ADP-induced binding to fibrinogen-coated beads), the VASP assay (quantification of phosphorylation of vasodilator-stimulated phosphoprotein), and a flow-cytometry-based assay (PACT) at four perioperative time points. Genotyping identified slow (CYP2C19*2 and CYP2C19*3) and fast (CYP2C19*17) metabolisers.

**Results:**

Between December 2017 and November 2019, 50 patients undergoing CEA were included. Platelet reactivity measured with the VerifyNow (p = < .001) and VASP (p = .029) changed over time, while the PACT did not. The VerifyNow identified patients changing HTRP status after surgery. The VASP identified patients changing HTPR status after eight weeks (p = .018). CYP2C19 genotyping identified 13 slow metabolisers.

**Conclusion:**

In patients undergoing CEA, perioperative platelet reactivity measurements fluctuate over time with little agreement between platelet reactivity assays. Consequently, HTPR status of individual patients measured with the VerifyNow and VASP assay changed over time. Therefore, generally used perioperative platelet reactivity measurements seem unreliable for adjusting perioperative APT strategy.

## Introduction

An estimated 40% of patients with coronary artery, cerebrovascular or peripheral arterial disease treated with clopidogrel have inadequate platelet inhibition. This phenomenon is called high on-treatment platelet reactivity (HTPR). Patients with HTPR have a 2.8-fold higher risk of cardiovascular events (CVE) compared to patients with adequate platelet inhibition [1]. Strategies to counter the thrombotic risk added by HTPR vary from changing the dosage and switching antiplatelet agents to combining antithrombotic drugs for a synergistic effect. Early identification of patients with HTPR may allow for timely medical intervention and improvement of the treatment decision process. Long-term platelet reactivity would preferably be equal or lower compared to pre-operative.

Platelet function tests (PFT) can be used to both identify HTPR and monitor thrombotic risk. The therapeutic platelet activity window consists of HTPR at one end of the spectrum and low platelet reactivity (LPR) at the other. Hypothetically, this allows for tailored antiplatelet therapy to lower residual platelet activity (e.g., a higher dose or different drug) to reduce thrombotic risk. However, currently, definitive conclusions cannot yet be drawn.

Polymorphisms of the CYP2C19 gene have been shown to influence the efficacy of several drugs, including clopidogrel [2]. CYP2C19 enzymes convert clopidogrel into its active metabolite. Common variants of the CYP2C19-gene result in slow metabolizer status, contributing to HTPR. Therefore, these patients are prone to a higher thrombotic risk. Both PFTs and genotyping have been proposed as tools to guide antiplatelet therapy. Personalized antiplatelet therapy based on platelet function testing in patients undergoing percutaneous coronary intervention proved non-inferior compared to genotype-guided APT [3–5].

A broad range of PFT assays are available, but the reproducibility, predictive value, and ease of use vary greatly [6]. Moreover, the optimal time for testing residual platelet activity is still unknown. Fundamental knowledge concerning the pharmacodynamics of antiplatelet therapy over time is needed to interpret PFT results. Therefore, we conducted the first study to assess individual perioperative platelet reactivity with various PFTs at multiple time points in patients undergoing carotid endarterectomy (CEA) and the influence of CYP2C19 polymorphisms on platelet activity.

## Methods

This pilot study was conducted at the University Medical Center Utrecht, The Netherlands, investigating perioperative platelet reactivity over time. This prospective single-centre observational cohort study explored the variability of platelet activity in patients undergoing CEA at four time points using the VerifyNow, VAsodilator-Stimulated Phosphoprotein (VASP) phosphorylation and the flow-cytometry-based platelet activation assay (PACT). Patients (≥ 18 years) were eligible if they were scheduled for an elective CEA, admitted to the hospital at least one day before and after surgery, and treated with the P2Y12-inhibitor clopidogrel. Patients were excluded if the surgical procedure exceeded 4 hours, required blood transfusion, postoperative intensive care admission was indicated, or treated with Vitamin-K antagonists or direct-acting oral anticoagulants (DOAC) before inclusion. The local ethics committee approved this study, and written informed consent was obtained from all patients following the Declaration of Helsinki.

All patients had started clopidogrel treatment (75mg q.d.) at least 24 hours before the intervention. Patients using acetylsalicylic acid were switched to clopidogrel (without a loading dose). Per the discretion of a stroke neurologist, patients who did not yet receive antiplatelet therapy at first presentation received a loading dose of 300mg clopidogrel at least 48 hours before the first measurement. All patients received clopidogrel 75mg q.d. during follow-up. Baseline characteristics were obtained from the electronic patient data file. All surgeries were performed by a vascular surgeon or a supervised vascular surgery fellow under general anaesthesia using longitudinal incisions. A fixed dose of 5000IU unfractionated heparin was administered peroperatively without reversion. Selective shunting was based on intraoperative neuromonitoring. All bedridden post-operative patients receive nadroparin (fraxiparin) injections.

The primary goal of this pilot study was to investigate individual fluctuation of perioperative platelet reactivity measured with platelet function tests. The secondary objective was to examine the influence of CYP2C19 polymorphisms on HTPR status.

### Sample collection & sample size

Platelet function was assessed at four time points: 1) before induction of anesthesia, 2) one day after surgery, 3) five days after surgery, and 4) at least eight weeks up to one year after surgery. The blood draws at each time point were considered to reflect 1) pre-operative platelet reactivity, 2) post-operative platelet reactivity (after tissue manipulation), 3) platelet reactivity in the postoperative (declining) phase, and 4) long-term stable platelet reactivity (on-treatment). A vacutainer containing Tri-sodium citrate 3.2% was used for all venous blood draws.

We calculated the sample size for this pilot study based on an extrapolation of the data from the only study investigating platelet reactivity variation in patients using P2Y12 inhibitors. Of all patients treated with 75mg clopidogrel, 41.4% (34.7–48.4%) showed a change in platelet reactivity of >40 PRU and 28.6% (22.6–35.2%) showed a change in platelet reactivity of >60 PRU measured with the VerifyNow as stated in an analysis of the ELEVATE-TIMI 56 Trial.^7^ The standard deviation was 68 PRU with a mean of 163.6 (SD 80.2). We calculated the sample size to assess the variation between two separate measurements. Assuming an intrapatient PRU-variation of 10% between test moments, we calculated a sample size of fifty patients using the 2-sample equivalence technique. The p-value was set at 0.05 and the power at 0.8, allowing statistical analysis of intra-test fluctuation between the time points and not the inter-test agreement. We provide insight into the effect of polymorphisms by showing trends within responder- and non-responder groups.

### Platelet function testing

Platelet reactivity was measured with the VerifyNow-P2Y12 assay according to the manufacturer’s instructions (Accumetrics, Inc.). A cut-off value of >40% inhibition or PRU <208 was regarded as adequate P2Y12 inhibition. HTPR status measured with the VerifyNow assay was defined as both >208 PRU and >230 PRU [7].

The VASP assay was performed according to the manufacturer’s instructions (Diagnostica Stago S.A.S.) [8]. Results were obtained by flow cytometry and expressed as platelet reactivity index (PRI). A cut-off value of PRI≥50% was used to define HTPR [9]. The blood was left for one hour before processing for the PACT assay. Five μL of whole blood was diluted 1:10 (v:v) in HEPES buffered saline (HBS; 10 mM HEPES, 150 mM NaCl, 1 mM MgSO4, 5 mM KCl, pH 7.4), containing 62.5 mM ADP and in‐house developed fluorophore-conjugated nanobodies against GPIbα (clone 17, APC) and fibrinogen (clone C3, Alexa488), or isotype control nanobody R2-Alexa488. Baseline platelet activation was assessed in the absence of a platelet agonist. Whole blood was incubated for 10 minutes at 37°C, after which samples were fixed with 0.148% formaldehyde, 137 mM NaCl, 2.7 mM KCl, 1.12 mM NaH2PO4, 10.2 mM Na2HPO4, 1.15 mM KH2PO4, 4 mM EDTA, pH 6.8 for 20 minutes at room temperature and analysed on a BD FACSCanto II (BD Biosciences). Platelets were identified based on forward and sideward scatter and GPIbα‐expression. Platelet fibrinogen binding was used as a marker of integrin aIIbβ3 activation. The flow cytometer was calibrated every week to maintain stable fluorescent intensity. All flow-cytometry data were corrected for baseline activation measured after stimulation with isotype control antibody (R2). Data are expressed as median fluorescence intensity (MFI). No HTPR cut-off value has been established for the PACT assay.

### Cyp2c19 genotyping

According to the manufacturer’s instructions, DNA for CYP2C19 genotyping was isolated from whole blood with the Gentra Puregene Blood Kit (Qiagen; Applied Biosystems, Foster City, CA, USA). The DNA was analysed for the presence of CYP2C19*2 (rs4244285), CYP2C19*3 (rs4986893) and CYP2C19*17 (rs12248560) polymorphisms with Taqman genotyping kits (Thermo-fisher scientific inc, Waltham, Massachusetts, United States) in a thermal cycling device (Biorad CFX96 Touch real-time PCR). The CYP2C1*2 single nucleotide polymorphism (SNP) allele rs4244285(A) is considered the most common variant, resulting in poor metabolism of clopidogrel (poor metabolisers). The CYP2C19*3 SNP allele rs4986893(A) is also thought to result in poor metabolising of clopidogrel. The CYP2C19*17 SNP, allele rs12248560(T), is associated with the ultra-rapid metabolisation of clopidogrel (fast metabolisers) [10]. Heterozygous or homozygous carriers of CYP2C19*2 and *3 variants were considered slow metabolisers, and heterozygous or homozygous CYP2C19*17 carriers were considered fast metabolisers. Homozygous and heterozygous carriers of the CYP2C19*2 allele were merged because both are considered poor metabolisers [11]. All others were considered wildtype.

### Statistical analysis

A paired t-test was used for normally distributed variables for continuous baseline variables and a Wilcoxon signed-rank test for non-normally distributed paired variables for all time points. We used a linear mixed model to analyse the predictive value of each test and time point (e.g., VerifyNow, PACT and VASP). We used a mixed-effects model to investigate the interaction between CYP2C19 polymorphisms and the PFT results. CYP2C19-polymorphisms (heterozygous and homozygous versus wildtype) were analysed by including them as the determinant of interest in the model. We tested the time by CYP2C19-polymorphism interaction. Model assumptions (i.e., distributional assumptions, homoscedasticity) were assessed with residual plots. The SAS statistical analysis system v9.4 was used for all analyses.

## Results

Between December 2017 and November 2019, 50 patients undergoing carotid endarterectomy were included. During the study period, two patients were prescribed DOACs by a cardiologist without consulting the study team and consequently lost to follow-up. The mean age of participants was 71.8 years, and 73% were male. The average BMI was 26.0 kg/m^2^. Lipid-lowering agents were used by 81% of patients, and beta-blockers were used by 35%. Diabetes Mellitus was present in 25% of the population, hypertension in 71% and kidney failure (defined as eGFR<30ml/min/1.72m^2^) in 17%. [Table 1]

**Table 1:** Baseline characteristics.

Baseline characteristics	N = (SD or %)
**Mean age (years)**	71,8 ± 9.2
**Sex (Male)**	35(73%)
**BMI (kg/m2)**	26.0 ± 3.9
**Smoking**	39(81%)
**Comorbidities (history of)**	
** • Diabetes Mellitus**	12(25)
** • Hypercholesterolemia**	30(63%)
** • Hypertension**	33(69%)
** • Kidney failure**	8(17%)
**Medication**	
** Clopidogrel**	
** • Clopidogrel 300mg**	7(14%)
** • Clopidogrel 75mg**	43(86%)
** • DOAC**	2(4%)
** Beta-blocker**	17(35%)
** Statin**	39(81%)
**CEA-side (left)**	20(42%)

*BMI body mass index **DOAC** direct-acting oral anticoagulant, prescribed after inclusion. **CEA** carotid endarterectomy **Kidney Failure** eGFR<30ml/min/1,72m^2^

Clopidogrel was prescribed to all patients. Seven patients (14%) received a clopidogrel loading dose of 300mg. Due to postoperative recovery time and logistic impairments, many patients could not visit the hospital after five days. Therefore, only nine patients are included for time point three.

### Platelet reactivity

**Fig 1:**
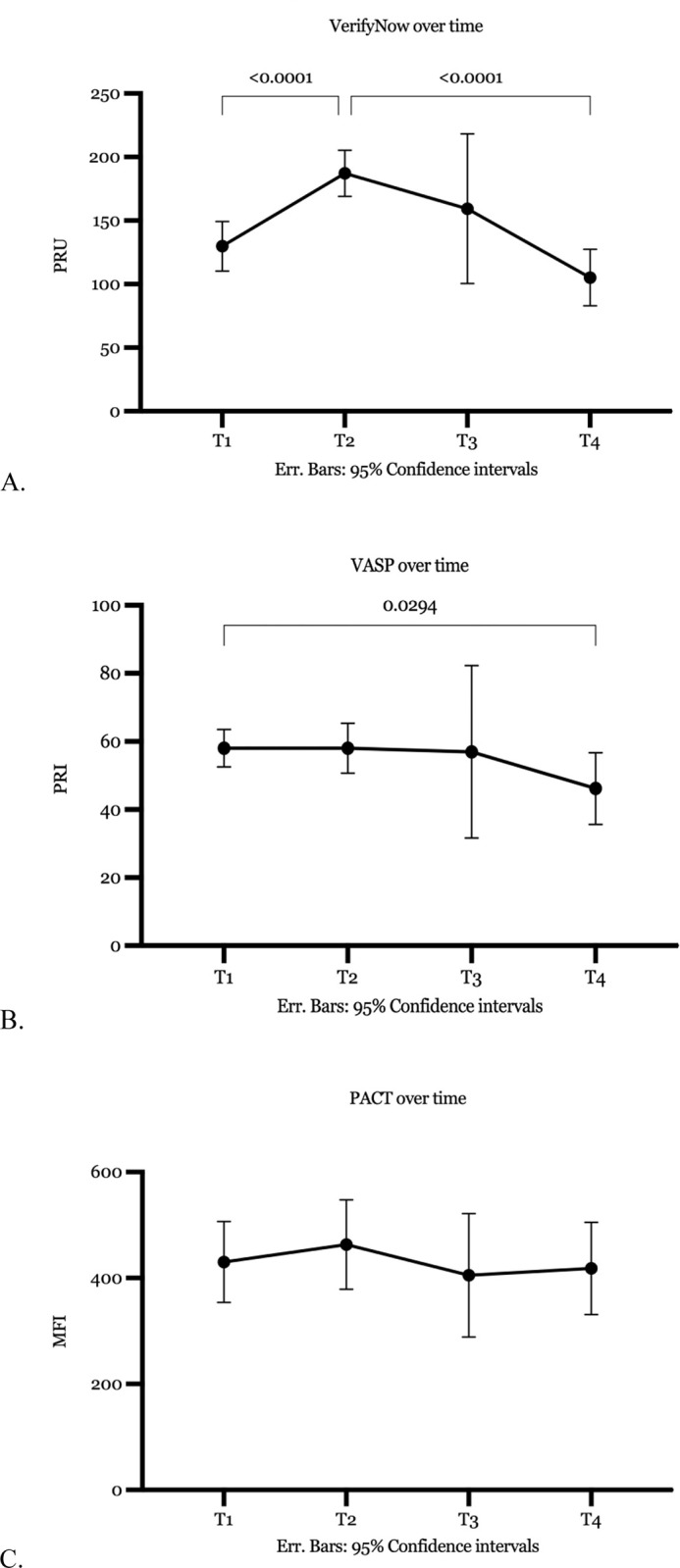
Platelet reactivity over time (A: VerifyNow, B VASP, C PACT). *PRU platelet-reactive units, * **VASP** vasodilator-stimulated phosphoprotein phosphorylation assay **PRI** platelet reactivity index, *PACT a flow-cytometry-based platelet function assay **MFI** median fluorescence intensity.

Platelet reactivity measured with the VerifyNow (p = < .001) and VASP (p = .029) changed over time, while platelet reactivity measured with the PACT did not. [Fig 1]. Platelet reactivity measured with VerifyNow was higher one day after surgery than at baseline or eight weeks after the procedure. This difference persisted when the patients who received a loading dose were excluded from the analysis. Baseline measurement results were similar to those eight weeks after the procedure.

In contrast, the procedure did not influence platelet reactivity measured with the VASP assay. However, platelet reactivity decreased eight weeks after surgery compared to baseline measurements. This difference persisted when the patients who received a loading dose were excluded from the analysis (p = .017).

### Htpr

HTPR status was defined for the VerifyNow and the VASP assay. [Table 2] 16 patients were identified with HTPR by the VerifyNow and the VASP at any time (not necessarily the same). According to VerifyNow, only two patients (4%) had HTPR at time point 4. One of these two patients had HTRP at all time points and was considered a “true non-responder”. The other was also diagnosed with HTPR at time point two. All others were considered “temporary HTPR patients”. Analysis using the more stringent cut-off value of >230 PRU resulted in 3 patients (6%) with HTPR before surgery,10 (20%) with HTPR after surgery (p = .007), and two (4%) with HTPR at least eight weeks after surgery. One of the patients identified with HTPR at time-point four was identified with HTRP at all time points. The other was not.

**Table 2:** Patients identified with HTPR per assay per time-point.

Assay	T1	*2 SNP	T2	*2 SNP	T3	*2 SNP	T4	*SNP
VerifyNow >208 PRU	8/50 (16%)	2/13	14/50 (28%)	4/13	2/7 (29%)	1/7	2/50 (4%)	1/13^±^
VerifyNow >230 PRU	3/50 (6%)	1/13	10/50 (20%)	3/13	2/7 (29%)	1/7	2/50 (4%)	1/13^±^
**VASP >50 PRI**	33/50 (66%)	11/13	35/50 (70%)	9/13	5/7 (71%)	1/7	18/40 (45%)	4/13
**VASP >60 PRI**	25/50 (50%)	9/13	23/50 (46%)	7/13	4/7 (57%)	1/7	16/40 (40%)	4/13

*HTPR High on-treatment platelet reactivity *2 SNP CYP2C19*2 polymorphism carriers **PRU** platelet-reactive units (cut-off value for HTPR oaf either 208 or 230 PRU) **VASP** vasodilator-stimulated phosphoprotein phosphorylation assay **PRI** platelet reactivity index (cut-off value for HTPR of either 50 or 60 PRI)

± Homozygous CYP2C19*2 carrier

Based on platelet function measured with the VASP assay, there were fewer patients with HTPR (PRI ≥ 50) at eight weeks after surgery than before surgery (p = .031) and compared with one day after surgery (p = .018). Eleven patients had HTPR at all time points. During the study period, there were no drug modifications in those with HTPR while on APT. The test results were blinded and did not lead to modifications in medication use during the study period. From all patients identified with HTPR at eight weeks, eight were CYP2C19-wildtype carriers, two were heterozygous carriers, and one was homozygous for a loss of function SNP (*2). A more stringent cut-off value for HTPR defined by the VASP of ≥60% has been proposed [12].

### Genotyping

CYP2C19 genotype analysis indicated that 13 patients were either homozygous or heterozygous carriers of the CYP2C19*2 SNP and were considered slow metabolisers. Twenty-four patients were either homozygous or heterozygous carriers of the CYP2C19*17 SNP and were considered fast metabolisers. [Table 3] Six patients were carriers of both a CYP2C19*2 and a CYP2C19*17 allele and were considered slow metabolisers.

**Table 3:** CYP2C19*2, CYP2C19*3 and CYP2C19*17 frequencies.

	Homozygous	Heterozygous	Wildtype	Total
Slow metabolisers				
CYP2c19*2	1 (2%)	12 (24%)	37 (74%)	50
CYP2c19*3	0	1 (2%)	0	1
Fast metabolizers				
CYP2C19*17	5 (10%)	19 (38%)	26 (52%)	50

*CYP2c19 cytochrome P450 2C19 enzyme protein CYP2C19*2 and CYP2C19*3 single-nucleotide polymorphism slow metaboliser variant CYP2C19*17 single-nucleotide polymorphism fast metaboliser variant

Two of 13 slow metaboliser SNP carriers had HTPR according to the VerifyNow before surgery (≥208PRU), which decreased to 1 out of 13 with the ≥230PRU cut-off value. Four out of 13 patients had HTPR one day after the procedure, and three out of 13 with the ≥230PRU cut-off value. Regardless of the cut-off value, only one patient had HTPR at all time points. This patient was homozygous for CYP2C19*2.

11 of 13 of the slow metaboliser SNP carriers had HTPR before surgery according to the VASP (PRI≥50). Nine out of 13 patients had HTPR one day after the procedure. Four patients had HTPR 8 weeks after surgery.

According to the VerifyNow (≥208PRU), four (16.6%) of the 24 fast metabolisers had HTPR before surgery, seven (29.2%) had HTPR one day after the procedure, and none had HTPR at eight weeks after surgery. When a more stringent cut-off was applied (≥230PRU), two (8.3%) had HTPR before surgery, four (16.6) had HTPR one day after surgery, and none had HTPR after eight weeks. None of them received a loading dose of clopidogrel.

According to the VASP assay, 16 (66.7%) of 24 fast metabolisers had HTPR (PRI≥50) before surgery. 18 (75%) had HTPR one day after the procedure, which decreased to five (20.8%) patients eight weeks after surgery (Fisher’s exact test p = .025).

## Discussion

This pilot study investigated perioperative fluctuation of individual platelet reactivity at four time points using three assays in patients undergoing CEA. We measured platelet reactivity before surgery, one day after surgery, five days after surgery and at least eight weeks up to one year postoperative (reflecting platelet reactivity during long-term treatment with clinically stable carotid artery disease). Our study has three notable findings: First, individual perioperative platelet reactivity measured with the VerifyNow and VASP assay fluctuated. Second, HTPR status measured with the VerifyNow and VASP assay changed over time. Third, although this study was not powered to analyse the effects of genotype on platelet reactivity, we found that at eight weeks after surgery, the fast metaboliser genotype carriers are less likely to be identified with HTPR according to the VASP assay.

To our knowledge, no data is available on the optimal moment to assess platelet reactivity and determine HTRP status. Most studies exploring the potential benefit of personalised APT have used a single platelet reactivity test for treatment stratification. Often, test results were obtained 12 to 24 hours after surgery.^4^ Although studies have demonstrated that the mean platelet activation across a population is consistent, individual fluctuation of platelet reactivity over time has been shown [13–16]. Thus far, only one post hoc analysis assessed platelet reactivity over time in individual patients. A sub-study of the ELEVATE-TIMI 56 (Escalating Clopidogrel by Involving a Genetic Strategy-Thrombolysis in Myocardial Infarction 56) trial evaluated platelet reactivity in patients using clopidogrel (75 or 150 mg) before and after 14 days of treatment^7^. Approximately one in every five patients changed responder status (ΔPRU >40, P<0.001) in individual analysis, indicating fluctuation in platelet reactivity even in patients with clinically stable cardiovascular disease.

Our study also showed fluctuation of perioperative platelet reactivity. The surgical procedure influenced VerifyNow measurements. Platelet reactivity was higher one day after surgery than before surgery. Also, the number of patients identified with HTPR changed directly after the procedure. Platelet reactivity and HTPR status measured with the VASP also fluctuated. The VASP showed lower platelet reactivity after eight weeks than before surgery and one day after. Postoperative platelet reactivity measurements with the VerifyNow assay carry a high risk of false-positive HTPR diagnoses. Previous studies based on postoperative PFT measurements to switch APT therapy may, therefore, be less reliable. Our data suggest that immediate postoperative assessment of platelet reactivity with the VerifyNow reflects a temporary elevation of platelet reactivity. As most studies investigating platelet function testing to guide antiplatelet therapy used postoperative VerifyNow measurements, the outcomes of these historical studies should be interpreted with caution.

Several studies on healthy subjects who did not receive APT have shown that platelet reactivity can vary significantly over time [17, 18]. This suggests that besides drug therapy (patho-)physiology, the patients’ diet, stress level, and pre-analytic variables such as concomitant medication and anaesthesia also affect platelet function over time [19]. In line with our findings, this supports the hypothesis that periprocedural platelet reactivity measurements are not unconditionally reliable. Beyond the scope of this pilot investigation, future studies should consider incorporating PPI usage data to elucidate potential interactions and provide a more nuanced analysis of perioperative platelet function in patients undergoing carotid endarterectomy.

In addition, diversity in baseline platelet reactivity resulting from carrying any of the various CYP2C19 gene pairs can influence individual thromboembolic risk. This does not obviate the urgency of finding the optimal time point for PFT assessment. Still, it might prove to be a tool to pre-select patients who benefit more from early identification of being non- or low-responsive to the prescribed platelet inhibitors.

Finally, this pilot study has not addressed drug-drug interactions such as that between P2Y12 inhibitors and proton pump inhibitors [20]. Future, focused studies are needed to investigate the influence of the abovementioned factors mitigating platelet function.

The VerifyNow assay showed fluctuation of platelet reactivity after surgery and after at least eight weeks compared to pre-operative and postoperative measurements, respectively. This substantiates the hypothesis that the surgical procedure influences platelet reactivity. Lower platelet reactivity and smaller proportions of patients identified with HTPR after eight weeks, shown by the VASP assay, also support this. The fluctuation of platelet reactivity might be caused by a prothrombotic state resulting from the surgical procedure itself. Consequently, it may be considered to intensify the perioperative antiplatelet regimen. Current guidelines suggest dual antiplatelet therapy (DAPT) for at least one month after stenting [21]. Measurements, therefore, should be taken either before or sufficiently long after the procedure to ensure that the intervention does not impact the measurement. Although, the exact timing is yet to be determined, platelet function testing might be a valuable tool for individualized (D)APT duration.

Implementing a period of intensive thrombotic risk reduction from the moment of diagnosis of carotid stenosis (e.g., after a CVA) could alleviate the added thrombotic risk of HTPR [22]. Potent agents are feasible, such as ticagrelor, DAPT, or direct-acting oral anticoagulants. The CAPRIE and recent COMPASS and VOYAGER trials have suggested intensifying antithrombotic therapy using either agents such as clopidogrel (CAPRIE) or adding low-dose direct-acting oral anticoagulants twice daily to platelet function inhibitors (acetylsalicylic acid plus rivaroxaban) [23–25]. The added risk of major bleeding appears low with short-term treatment, though cost-effectiveness needs to be proven. After surgery, repeated PFTs at a regulated interval could guide phased downscaling of APT to an acceptable level within the therapeutic window between thrombotic risk and risk of bleeding. Platelet function assays must overcome their limitations for applicability in daily practice. Further research focussing on patients with carotid artery disease is needed to investigate the effect of this strategy on outcome.

## Conclusion

In patients undergoing CEA, perioperative platelet reactivity measurements fluctuate over time. Additionally, the HTPR status of the individual patient, as measured with the VerifyNow and VASP assay, changed over time. Therefore, commonly available perioperative platelet reactivity measurements are unreliable for perioperative APT strategy adjustments.

## Supporting information

S1 File This excel file contains the complete dataset used. (XLSX)
